# Full-ring Intrastromal Corneal Implantation for Correcting High Myopia in Patients with Severe Keratoconus

**Published:** 2016

**Authors:** Khosrow JADIDI, Farhad NEJAT, Seyedali Asghar MOSAVI, Mostafa NADERI, Ali KATIRAEE, Leila JANANI, Hossein AGHAMOLLAEI

**Affiliations:** 1 Department of Ophthalmology, Baqiyatallah University of Medical Sciences, Tehran, Iran; 2 Bina Eye Hospital, Tehran, Iran; 3 Department of Biostatistics, School of Public Health, Iran University of Medical Sciences, Tehran, Iran; 4 Young Researchers and Elites Club, North Tehran Branch, Islamic Azad University, Tehran, Iran

**Keywords:** Full-ring Intrastromal Corneal Ring, High Myopia, Severe Keratoconus, MyoRing

## Abstract

The aim of this study was to evaluate the effect of the mechanical implantation of a MyoRing in patients with severe keratoconus and high myopia. The study involved 32 eyes of 32 patients (14 men and 18 women; mean age: 29.6 ± 6.7; age range: 20 – 44). The patients underwent MyoRing implantation with mechanical dissection using a Pocket Maker microkeratome, and outcomes were assessedat3 months after surgery. The main outcome measures were uncorrected and corrected distance visual acuity (UDVA and CDVA, both in Logarithm of the Minimum Angle of Resolution [logMAR] units), manifest refraction, and keratometry readings. There was a significant improvement in the UDVA, from 1.14 ± 0.32 to 0.35 ± 0.24 (P ˂ 0.001), and in the CDVA, from 0.47 ± 0.20 to 0.22 ± 0.15 (P ˂ 0.001). There was also a significant improvement in the spherical equivalent refractive error (-10.51 ± 2.81 D to -1.32 ± 2.29 D) (P ˂ 0.001). There was a significant decrease of manifest refraction in the mean sphere and cylinder of 7.70 and 2.6 D, respectively (P < 0.001). Furthermore, with regard to corneal topography, there was a significant reduction of 3.55 D (P ˂ 0.001) in the mean keratometry reading. The results show that the mechanical implantation of a MyoRing is effective for the correction of myopia in patients with keratoconus and high myopia.

## Introduction

Keratoconus is a non-inflammatory progressive corneal ectatic disorder that is often bilateral (though asymmetrical) and can induce irregular astigmatism with or without myopia (1, 2). It can cause mild to severe vision impairment. The prevalence of keratoconus in Iran is reported to be 0.76 – 3.3%, which is higher than the global average (3, 4). Various treatments have been used for the disease. Traditionally, glasses and contact lenses were used in the early stages of the disease, and corneal transplants were used in severe to advanced cases (5, 6). However, corneal transplants have limitations(7). In recent years, new surgical alternatives have been developed. For example, collagen cross-linking can be used to strengthen the cornea and prevent progression of the disease (8, 9). In addition, intrastromal corneal ring segments can be used to decrease astigmatism through an arc-shortening effect of the corneal lamellae that produces a flattening the central cornea (10, 11). The advantages of using ring segments within the corneal stroma include safety, reversibility, and stability without impairment of the optic axis (12, 13).

Myopia is one of the most common manifestations of keratoconus. Usually, the severity of myopia is related to the severity of keratoconus. However, the usual treatments for myopia, such as photorefractive keratectomy (14), epikeratophakia, (15), and laser-assisted in situ keratomileusis (16), are not suitable for patients with keratoconus. Moreover, there is a new treatment option that involves using a corneal intrastromal implantation system (CISIS). This treatment involves a full-ring flexible implant known as a MyoRing (DIOPTEX GmbH, Linz, Austria) that is implanted into a corneal pocket. It is not only effective for treating keratoconus, but can also be effective for treating moderate to high myopia (17-20). As there have been no studies on Iranian patients with keratoconus and high myopia, this study was performed to evaluate the effectiveness of the MyoRing implantation on visual acuity and refraction in patients with keratoconus and high myopia in Iran.

## METHODS

Study Subjects

The study involved 32 eyes of 32 patients with keratoconus and high myopia who underwent MyoRing implantation in 2015 at Bina Eye Hospital, Tehran, Iran. Diagnosis of keratoconus was established (using an Orbscan IIz Topographer, Bausch & Lomb, Claremont, CA) by a combination of computerized videokeratography readings of the anterior and posterior corneal surfaces and corneal pachymetry. High myopia was defined as myopia ≥ 6.00 D. After the purpose of the study and the procedures that would be involved were fully explained, all patients were asked to sign an informed consent form before being enrolled into the study. The inclusion criteria were the presence of poor visual acuity with glasses, contact lens intolerance, a clear central cornea, a minimum corneal thickness of 360 µm (19, 21, 22), and a mean keratometry reading of 45 – 52 D. The exclusion criteria were a positive pregnancy test, breastfeeding, use of immunosuppressive drugs, previous keratorefractive surgery on the eye to be operated on, having a history of vernal or atopic keratoconjunctivitis or a corneal stromal disorder, and having dry eye syndrome, nystagmus, hyperopia, and a severe ocular (e.g., herpes keratitis, glaucoma, cataracts, diabetic retinopathy, age-related macular degeneration) or systemic disease (e.g., an autoimmune disease or a systemic connective tissue disease).

Assessments

The preoperative assessments involved UDVA, CDVA, and manifest refraction assessments and keratometry readings. The same assessments were carried out at 3 months after surgery. Visual acuity was measured using a Snellen chart, and transformed into a Logarithm of the Minimum Angle of Resolution (logMAR) value for statistical analysis, and corneal topography was measured using the Orbscan IIz Topographer.

Surgical Procedure

**Figure 1:**
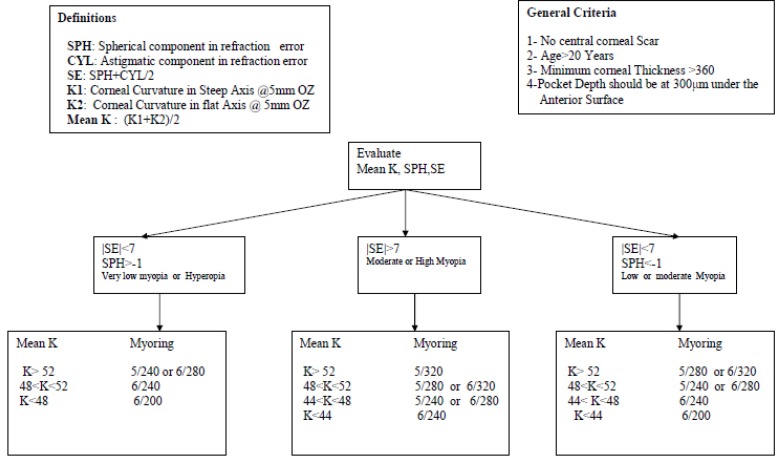
Guidelines for the selection of MyoRings for patients with keratoconus

The same surgeon (KHJ) performed all the surgeries under sterile conditions with topical anesthesia (0.5% proparacaine hydrochloride solution). The appropriate MyoRing diameter (5- or 6-mm diameter) and thickness was selected in accordance with innovative guidelines developed based on the authors’ experiences (Fig. 1), and then the MyoRing was implanted into the eye.

The use of the Pocket Maker microkeratome (DIOPTEX GmbH) has been described in detail previously (23, 24). In brief, an intrastromal pocket, 9 mm in diameter and 300 µm in depth, was created using a small incision. The MyoRing implant was then placed into the pocket using implantation forceps, and its position was adjusted using a keratoscope. The pocket was self-sealing and did not require suturing (21). No intraoperative complications occurred. Subsequently, a PureVision silicone hydrogel bandage contact lens (Bausch & Lomb) was placed on the cornea and then removed 1 day after the surgery. Postoperative treatment comprised betamethasone drops (Sina Darou Laboratories, Tehran, Iran) four times a day, chloramphenicol drops (Sina Darou Laboratories, Tehran, Iran) four times a day, and preservative-free artificial tears (Artelac Rebalance, Bausch & Lomb, Inc., North Bridgewater, NJ, USA) six times a day. The chloramphenicol drops were discontinued 1 week after the surgery, while the betamethasone dosage was tapered over a period of 4 to 6 weeks.

Statistical Analysis

The categorical variables (sex and number of right and left eyes) are expressed as frequencies (with percentages). The continuous variables (age and the pre- and postoperative UDVA, CDVA, sphere, cylinder, spherical equivalent, K_max_, K_min_, and K_mean _measurements) are expressed as means (with standard deviations). The differences between the preoperative and postoperative measurements (i.e., UDVA, CDVA, sphere, cylinder, spherical equivalent, K_max_, K_min_, and K_mean _measurements) were compared using paired t-tests. For the between-group analysis (which compared the outcomes of the patients treated with 5- versus 6-mm-diameter MyoRings), the continuous variables (UDVA, CDVA, sphere, cylinder, and spherical equivalent) are expressed as medians (with 25th and 75th percentiles), and the between-group differences were compared using Mann–Whitney U tests. The statistical analysis was performed using SPSS for Windows (version 18; SPSS Inc., Chicago, IL), and p values < 0.05 were considered statistically significant.

## RESULTS

The study was conducted on 14 men (43.8%) and 18 women (56.2%). The mean age was 29.6 ± 6.7 years (range: 20 – 44) (Table 1). Of the 32 participants, 19 had keratoconus in the left eye, and 13 had keratoconus in the right eye (Table 1).

**Table 1:** Characteristics of Participants, No. = 32

**Variable**	**Values**
**Gender**	
**Male**	14 (43.8%)
**Female**	18 (56.3 %)
**Age**	29.6 ± 6.7
**Range**	20–44
**Number of eyes**	
**Right**	13 (40.62)
**Left**	19 (59.37)

SD: standard deviation.

Data in table are presented as No. (%) or Mean ± SD

The mean pre- and postoperative UDVA, CDVA, spherical equivalent, cylinder, sphere, and keratometry readings are presented in Table 2. The mean preoperative UDVA was 1.14 ± 0.32 logMAR, which improved to 0.35 ± 0.24 logMAR at 3 months after surgery (< 0.001), and the mean preoperative CDVA was 0.47 ± 0.20 logMAR, which improved to 0.22 ± 0.15 logMAR after surgery (< 0.001). The mean preoperative spherical equivalent was -10.51 ± 2.81 D, which decreased to -1.32 ± 2.29 D after surgery. The mean preoperative cylinder was -4.59 ± 1.86 D, which decreased to -2.00 ± 1.51 D after surgery. In particular, the mean sphere was considerably reduced by -0.32 ± 2.15 D from -8.18 ± 2.58 D. Furthermore, the mean keratometry reading was 51.06 ± 3.26 D, which decreased to 47.51 ± 3.57 D after surgery. 

**Table 2:** Comparisons of Pre- and Postoperative Visual Acuity, Refractive and keratometric variables

**Variable**	**Preoperative values**	**Postoperative values**	**P value**
**UDVA (logMAR)**			
Mean ± SD	1.14 ± 0.32	0.35 ± 0.24	< 0.001
Range	0.40, 1.6	0.0, 1.0	
**CDVA (logMAR)**			
Mean ± SD	0.47 ± 0.20	0.22 ± 0.15	< 0.001
Range	0.1, 1.0	0.0, 0.7	
**Sphere(D)**			
Mean ± SD	-8.18 ± 2.58	-0.32 ± 2.15	< 0.001
Range	-15, -6	-5.5, 3.5	
**Cylinder(D)**			
Mean ± SD	-4.59 ± 1.86	-2.00 ± 1.51	< 0.001
Range	-9.5, -0.6	-5.0, 1.5	
**Spherical equivalent(D)**			
Mean ± SD	-10.51 ± 2.81	-1.32 ± 2.29	< 0.001
Range	-17.37, -6.3	-7.0, 2.25	
**K**_max_ **(D)**	53.62 ± 3.57	48.96 ± 3.55	< 0.001
**K**_min_ **(D)**	48.58 ± 3.16	46.15 ± 3.78	< 0.001
**K**_mean_ **(D)**	51.06 ± 3.26	47.51 ± 3.57	< 0.001

UDVA: uncorrected distance visual acuity, logMAR: Logarithm of the Minimum Angle of Resolution, CDVA: corrected distance visual acuity, D: diopter, K_max_: maximum keratometry value in D, K_min_: minimum keratometry value in D, K_mean_: mean keratometry value in D. Based on the previously mentioned guidelines, a 5-mm-diameter MyoRing was used for 21 eyes (65.6%) and a 6-mm-diameter MyoRing was used for the remaining 11 eyes. The between-group differences in the median changes at 3 months after surgery in sphere and spherical equivalent were significant (Table 3). The median change in sphere for the 5- and 6-mm-diameter MyoRing groups was 8.5 and 6 D, respectively (P = 0.027), and the median change in spherical equivalent for the 5- and 6-mm-diameter MyoRing groups was 10 and 7 D, respectively (P = 0.027). However, the between-group differences in the median changes in UDVA, CDVA, and cylinder were not significant (Table 3).

**Table 3:** Comparison of visual acuity and refractive variables between the 5- and 6-mm-diameter MyoRing groups

**MyoRing diameter(mm)**	**Median (25th and 75th percentiles)**	**P value**
**Spherical equivalent(D)**		0.024
5	10 (7.08, 12.25)	
6	7 (6.0,9.25)	
**UDVA (logMAR)**		0.968
5	-0.90 (-1.15, -0.3)	
6	-0.85 (1.00,0.6)	
**CDVA (logMAR)**		0.216
5	-0.3 (-0.4, -0.1)	
6	0.25 (0.3, 0.0)	
**Sphere (D)**		0.027
5	8.5 (6.5, 10.37)	
6	6 (5.25, 7.5)	
**Cylinder (D)**		0.750
5	3 (0.87, 4.62)	
6	2 (1.00, 3.50)	

UDVA: uncorrected distance visual acuity, logMAR: Logarithm of the Minimum Angle of Resolution, CDVA: corrected distance visual acuity, D: diopter

## DISCUSSION

This study evaluated the effect of MyoRing implantation in patients with keratoconus and high myopia 3 months after surgery. We found a remarkable improvement in the UDVA, CDVA, spherical equivalent, sphere, and cylinder, and the postoperative sphere was considerably reduced. We believe that these positive outcomes were partly the result of our newly developed guidelines for the selection of MyoRings for patients with keratoconus (Fig. 1). The improvements indicate that MyoRing implantation is an effective method for treating high myopia. The degree of reduction in the sphere was consistent with that observed in a similar study by Daxer et al. (25), which showed that there was a reduction in the sphere from -5.13 ± 4.34 D to 0.10 ± 3.2 D at 1 year after MyoRing implantation. In the study by Daxer *et al*., the mean CDVA and UDVA improved from 0.42 (0.40 + - 0.17 logMAR) to 0.77 (0.12 + - 0.10 logMAR), and from 0.07 (1.24 +/- 0.35 logMAR) to 0.56 (0.27 + - 0.17 logMAR), respectively. This change in the mean CDVA was greater than that observed in our study (from 0.47 to 0.22 logMAR), which could be due to the shorter follow-up period in our study. However, previous research on the implantation of intrastromal corneal ring segments indicated that here was stability in the refraction and visual acuity outcomes between 3 and 6 months after surgery (26, 27). Nevertheless, there are significant differences in treatment-related biomechanics between intrastromal corneal ring segments, incomplete rings, and complete rings (such as the MyoRing) (28).

The reduction in the mean keratometry value at 3 months after surgery was statistically significant, which is consistent with the results of the study by Daxer et al., who observed a significant reduction in the mean keratometry value of 48.96 to 43.20 D (25). The spherical equivalent in their study improved from -6.27 ± 5.20 D to -0.52 ± 3.4D (25), while we observed a decrease in the spherical equivalent from -10.51 ± 2.81 D to -1.32 ± 2.29 D after surgery. Furthermore, we observed a reduction in the cylinder of approximately 2.6D after surgery. Similarly, the study by Daxer et al. showed that there was a reduction in the cylinder from -3.50 to -1.27 D (25). In addition, we assessed the influence of the diameter of the MyoRing on the outcomes. We found that there was a significant relationship between the diameter and the median changes in sphere and spherical equivalent, with the 5-mm-diameter MyoRing being more effective than the 6-mm-diameter MyoRing. However, the baseline characteristics of the patients in the 5- and 6-mm-diameter groups were not identical (as they were non-randomly assigned to the two groups based on the factors shown in Fig. 1), so these results are not conclusive. A potential limitation of our study is the short follow-up period. Further studies with longer follow-up periods and multi-center collaboration are recommended to allow firmer conclusions to be drawn. MyoRing implantation not only reduces myopia in patients with keratoconus and high myopia, but it also improves UDVA, CDVA, spherical equivalent, sphere, cylinder, and keratometry readings. MyoRing implantation can be considered a safe and effective treatment for patients with keratoconus and high myopia.
